# Relationships Between Markers of Iron Status and Hematological Parameters in Patients With Sickle Cell Disease

**DOI:** 10.1155/ah/9872440

**Published:** 2024-12-03

**Authors:** Nermi L. Parrow, Jason M. Doherty, Anna Conrey, Swee Lay Thein, Robert E. Fleming

**Affiliations:** ^1^Department of Pediatrics, Saint Louis University School of Medicine, St. Louis, Missouri, USA; ^2^Advanced HEAlth Data (AHEAD) Research Institute, Saint Louis University School of Medicine, St. Louis, Missouri, USA; ^3^Sickle Cell Branch, National Heart, Lung and Blood Institute, National Institutes of Health, Bethesda, Maryland, USA; ^4^Edward A. Doisy Department of Biochemistry and Molecular Biology, Saint Louis University School of Medicine, St. Louis, Missouri, USA

**Keywords:** iron, mean cellular hemoglobin concentration, sickle cell disease, transferrin saturation

## Abstract

Based on the relationship between the intracellular concentration of sickle hemoglobin S (HbS) and the delay that occurs prior to the onset of sickling following deoxygenation, targeting the intracellular HbS concentration is a recognized therapeutic approach for sickle cell disease (SCD). We and others have shown that restricting iron by dietary or pharmacologic means improves hematologic parameters, inflammation, and organ damage in mouse models of SCD. Clinical evidence corroborating these findings is confined to case reports and small case series studies, none of which account for treatment or *α*-thalassemia. We hypothesize that increased transferrin saturation is associated with increased mean cellular hemoglobin concentration (MCHC) which in turn is associated with decreased red cell counts and worsening anemia. To investigate this hypothesis, we examined the relationships between transferrin saturation and MCHC with each of the parameters that define MCHC in sickle patients (HbSS without *α*-thalassemia) and healthy volunteers (HVs). Results indicate that transferrin saturation and MCHC are positively correlated with each other in sickle patients and HV. In patients with SCD, MCHC and transferrin saturation are negatively correlated with RBC count and are not correlated with hemoglobin, whereas each is positively associated with HV. Transferrin saturation and MCHC are each positively correlated with the hemolysis marker, lactate dehydrogenase. These observations support a model where increased transferrin saturation contributes to higher intracellular HbS concentrations with subsequent increases in sickling and hemolysis in sickle patients, suggesting that pharmacologic approaches to decrease serum iron may provide a therapeutic approach for patients with SCD.

**Trial Registration:** This study was registered with ClinicalTrials.gov identifiers: NCT00011648, NCT00081523, and NCT04817670.

**Summary**

• Transferrin saturation is positively correlated with MCHC.
• Hemoglobin is positively associated with MCHC and transferrin saturation in HVs but not in SCD patients.
• RBC counts are negatively associated with MCHC and transferrin saturation in SCD patients but not associated with MCHC in HVs.
• These differences in the relationships between iron status and hematological parameters in sickle patients suggest that pharmacologic approaches to decrease serum iron may provide a therapeutic approach for patients with SCD.

## 1. Introduction

Polymerization of hemoglobin S (HbS) leads to the altered shape, function, and integrity of erythrocytes in patients with sickle cell disease (SCD), initiating the inflammation and oxidative stress that underly the pathophysiology. Several factors modulate disease severity, including coinheritance of *α*-thalassemia, glucose-6-phosphate dehydrogenase levels, and iron status [1, 2]. Sickling is initiated upon erythrocyte exposure to the decreased oxygen tensions in the microvasculature, causing the HbS strands to align, polymerize, and distort the cell membrane [3]. Importantly, polymerization does not immediately follow deoxygenation but is delayed over a timeframe that is inversely proportional to the ∼30^th^ power of the erythrocyte hemoglobin concentration [4]. This observation has led to the “delay time hypothesis,” which proposes that erythrocytes with lower hemoglobin (HbS) concentrations will be slower to sickle, and thus more likely to traverse the hypoxic microvasculature unaffected [5]. *Ex vivo* studies demonstrate that inducing cellular dehydration increases the mean cellular hemoglobin concentration (MCHC) and the propensity of the cells to sickle. In SCD patients, functional abnormalities in membrane ion channels result in erythrocyte dehydration, a smaller MCV, and a greater MCHC. As such, patients with more “dense” erythrocytes have worse hemolysis and attendant complications.

Case reports and studies in murine models of SCD identify iron restriction as a possible means by which erythroid parameters can be improved. In the Townes murine model of SCD, modest dietary iron restriction decreases the MCHC, decreases the propensity to sickle, improves the RBC count [6], and protects against vaso-occlusion [7]. Iron-restricted erythropoiesis mediated by pharmacologic inhibition of the iron exporter ferroportin decreases the adhesion of erythrocytes to the vascular endothelium and likewise reduces markers of hemolysis in the Townes murine model of SCD [8]. In a marrow transplant model of SCD, iron restriction (caused by genetic disruption of intestinal hypoxia-inducible factor-*α*) is associated with improved hematologic parameters and prolonged erythrocyte survival [9]. To date, reported findings in human patients with SCD are limited to isolated case reports and small case series; however, they suggest an association between iron deficiency and improved hematologic and clinical parameters [10–12].

Based on these observations, we hypothesized that increased saturation of plasma transferrin with iron is associated with increased MCHC. We further hypothesize that MCHC, in turn, is associated with a decreased RBC count and worse anemia. Since MCHC is determined by hemoglobin, RBC number, and mean cell volume (MCV), we investigated the relationship of transferrin saturation and MCHC with each of these parameters in patients with SCD and healthy volunteers (HVs).

## 2. Methods

### 2.1. Patient Data

We utilized a database consisting of 795 patients with SCD (including different sickle genotypes) seen at the National Institutes of Health (NIH) between 2001 and 2015. These data were obtained from patients who provided written informed consent in accordance with the Declaration of Helsinki and National Heart, Lung, and Blood Institute Institutional Review Board–approved protocols. Depending on the date of the patient visit, complete blood counts were performed by the NIH clinical laboratory using either CELL-DYN 4000 (through ∼ 2007), Cell-Dyne Sapphire (∼2007–2009), Sysmex XE-500 (2009), or Sysmex XN (∼2009–2015) hematology analyzers. Data were filtered based on sickle genotype, *α*-thalassemia genotype, hydroxyurea treatment, transfusion status, and availability of serum iron and hematologic parameters within a single visit. The primary analyses were confined to SCD patients with HbSS genotype, without *α*-globin triplication or deletion, not on hydroxyurea, and without laboratory evidence of recent transfusion (determined by summed HbS, HbA_2_, and HbF ≥ 99.9% and absence of HbA, Supporting Figure 1). We additionally analyzed selected markers of hemolysis, including serum lactate dehydrogenase [13]. Complementary analyses were performed on SCD patients with HbSS genotype who were taking hydroxyurea but without *α*-globin triplication or deletion and without evidence of recent transfusion. Data from HVs were obtained from the NIH Biomedical Translational Research Information System (BTRIS) database, filtering for those without detectable HbS that had hematologic and iron panel data obtained on the same visit and matched in terms of gender, race, and age range. Subjects with overt iron deficiency, as evidenced by a Mentzer index <13, [14] or statistical outlier MCV value were excluded.

### 2.2. Statistical Analyses

For multiple regression analyses, data were analyzed in R [15] via multiple linear regression models for normal and near-normal distributed outcome variables, as our sample size is sufficiently high that mild skewness of outcome variables is unlikely to affect model fit [16–19]. Transferrin saturation was found to be more heavily right-skewed than other variables, while analyzing the data via linear regression violated the assumption of homoscedasticity. No other models violated this assumption. Therefore, the transferrin saturation analyses were conducted with inverse gamma generalized linear regression modeling. Interaction terms were included to test differences in biomarker relationships between groups, and the inclusion of these interactions was assessed via a comparison of the Akaike and Bayesian information criteria (AIC and BIC). For all analyses except the analysis of MCHC and Hb, AICs and BICs supported the inclusion of the interaction effects (data not shown). For the MCHC and RBC analysis, while AIC supported the inclusion of the interaction effect, BIC comparison indicated no evidence for or against the inclusion of the interaction (Bayes factor = 1.00): considering this mixed evidence, we elect to report the full model to retain consistency with the other analyses. To provide evidence for changes in slope magnitude and direction, group slopes were estimated using the *marginaleffects* R package [20] to calculate a partial derivative for the variables of interest. Simple linear associations were analyzed by the Pearson correlation coefficient, *r*, in GraphPad Prism 10.0.

Scatter plots and the heat map were analyzed in GraphPad Prism 10.0. Statistical significance was analyzed by one-way ANOVA, with *p* values < 0.05 considered as statistically significant.

## 3. Results

### 3.1. Baseline Characteristics Demonstrate the Expected Differences Between HVs and Patients With SCD

To investigate the relationships between transferrin saturation, MCHC, and anemia, we utilized a database of patients with SCD seen at the NIH between 2001 and 2015. From that database, patients with a HbSS genotype who did not have *α*-thalassemia or evidence of recent blood transfusion and who had relevant hematologic and/or iron panels obtained on the same visit were stratified by hydroxyurea status for analysis (Supporting Figure 1). For comparison, the NIH BTRIS was mined for HVs similar in terms of age, race, and gender, without detectable HbS and who also had relevant hematologic and/or iron panels obtained on the same visit. Patients with SCD ranged from 18 to 82 years of age and HVs ranged from 15 to 77 years of age. Baseline characteristics are summarized in Supporting Table 1. Initial analyses of hematologic parameters demonstrate the expected decreases in hemoglobin, RBC counts, and hematocrit in patients with SCD compared to HVs (Supporting Figures 2A–2C). Hemoglobin and hematocrit are increased in patients taking hydroxyurea compared to those who were not, but no significant differences are observed in RBC counts between these cohorts. Mean cellular hemoglobin (MCH) is increased in SCD patients compared to HVs and is significantly higher in SCD patients on hydroxyurea compared to SCD patients not taking the drug (Supporting Figure 2D). Mean cellular volume (MCV) is increased in SCD patients taking hydroxyurea compared to SCD disease patients not taking the drug and HVs. No significant differences in MCV are observed between the latter two groups (Supporting Figure 2E). MCHC is increased in patients with SCD compared to HVs irrespective of hydroxyurea status and no differences are observed between SCD patients with or without hydroxyurea (Supporting Figure 2F).

Transferrin saturation was evaluated as an indicator of iron status. Data demonstrate higher transferrin saturation in both cohorts of patients with SCD compared to HVs (Supporting Figure 3). Transferrin saturations are significantly higher in SCD patients taking hydroxyurea than in SCD patients not taking the drug.

Differences in fetal hemoglobin were evaluated between the SCD patients as a function of hydroxyurea status and the data demonstrate the expected increase in fetal hemoglobin in SCD patients taking hydroxyurea compared to those not on the drug (Supporting Figure 4A). The percentage of reticulocytes is lower in SCD patients taking hydroxyurea compared to those who are not (Supporting Figure 4B), consistent with an improvement in anemia. Lactate dehydrogenase, a marker of hemolysis, is likewise lower in SCD patients taking hydroxyurea compared to those who are not (Supporting Figure 4C). Overall, these data demonstrate the ranges in hematologic and transferrin parameters between each cohort and confirm the expected effects of hydroxyurea treatment, including improved hematocrit, increased MCH and MCV [21], higher fetal hemoglobin concentration, decreased reticulocyte percentage, and decreased lactate dehydrogenase.

### 3.2. The Relationship Between Transferrin Saturation and Selected Hematological Parameters Is Altered in Patients With SCD Compared to HVs

**Figure 1:**
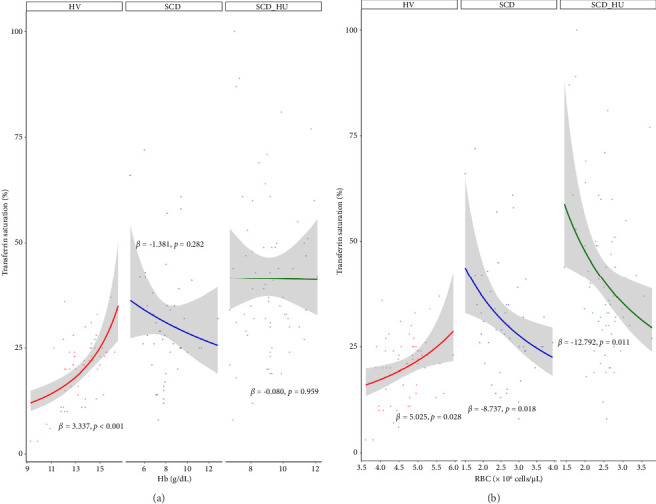
Relationship between transferrin saturation and hematological parameters across groups. Regression analyses demonstrate the relationships between transferrin saturation and (a) hemoglobin and (b) RBC counts in healthy volunteers (*n* = 58), untreated sickle cell disease patients (*n* = 48), and sickle cell disease patients receiving hydroxyurea(*n* = 67). Annotated slope estimates are taken from Table 2.

**Figure 2:**
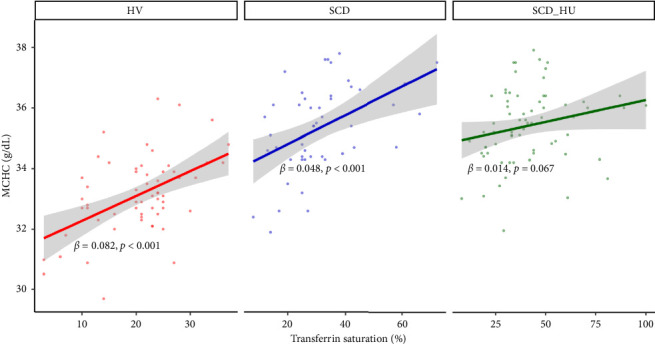
Relationships between mean cellular hemoglobin concentration and transferrin saturation across groups. Regression analyses demonstrate the relationships between mean cellular hemoglobin concentration in healthy volunteers (*n* = 58), untreated sickle cell disease patients (*n* = 48), and sickle cell disease patients receiving hydroxyurea (*n* = 67). Annotated slope estimates are taken from Table 2.

With the baseline differences in hematologic and iron parameters established, we focused on the relationship between transferrin saturation and the hematologic parameters that determine MCHC, primarily hemoglobin and RBC count. Analyses compared data from patients with SCD to those obtained from HVs. Patients with SCD were stratified based on hydroxyurea status. Transferrin saturation has a positive association with both hemoglobin and RBC count in HVs (Figures 1(a) and 1(b): see statistically significant positive slope for HV in 1a and 1b; Table 1, Models 3 and 4: see statistically significant positive coefficients for Hb and for RBC, both estimated on the control group of HVs). In SCD patients who are not on hydroxyurea, transferrin saturation is not associated with hemoglobin concentration (Figure 1(a): see nonsignificant slope estimate for SCD group; Table 1: see statistically significant interaction effect for the SCD group; Table 2: see slope estimate for the SCD group) and has an inverse relationship with RBC counts (Figure 1(b): see statistically significant negative slope for the SCD group), as indicated by the significant interaction shown in Table 1 and the negative slope estimate (Table 2). The same altered relationships were observed in patients with SCD on hydroxyurea (Figures 1(a) and 1(b); Tables 1 and 2). Transferrin saturation has a positive relationship with MCV in HVs and in patients with SCD, independent of hydroxyurea status (data not shown). Likewise, transferrin saturation has a positive relationship with MCHC in HVs and in patients with SCD, although it is not statistically significant in SCD patients taking hydroxyurea (*p* = 0.067) likely due to increased scatter (Figure 2 and Table 3). Slope estimates, providing the direction and strength of the correlations that describe the relationships between transferrin saturation and hemoglobin and RBC count, confirm and quantify the differences in HVs and patients with SCD, regardless of hydroxyurea status (Table 2). No significant differences between the two groups are observed in the correlation coefficients that describe the relationship between transferrin saturation and MCV and MCHC. Of note, no relationship between transferrin saturation and fetal hemoglobin is evident (Supporting Figure 5). There is also no significant relationship between hematological parameters and ferritin in patients with HbSS (data not shown), likely because ferritin, a highly sensitive acute phase reactant, is more reflective of inflammation than iron status in this setting.

### 3.3. The Relationship Between MCHC and Selected Hematological Parameters Is Altered in Patients With SCD Compared to HVs

**Figure 3:**
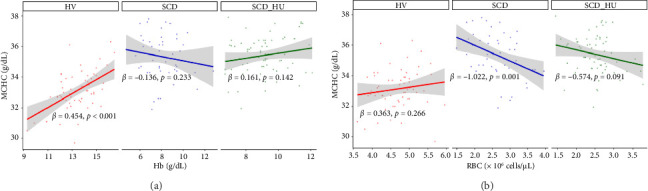
Relationship between mean cellular hemoglobin concentration and hematological parameters across groups. Regression analyses demonstrate the relationships between MCHC and (a) hemoglobin and (b) RBC counts in healthy volunteers (*n* = 58), untreated sickle cell disease patients (*n* = 48), and sickle cell disease patients receiving hydroxyurea (*n* = 67). Annotated slope estimates are taken from Table 2.

To investigate the hypothesis that increased MCHC is associated with a more profound anemia in patients with SCD, we examined the relationships between MCHC and hemoglobin and RBC count in patients with SCD and HVs. Results demonstrate that hemoglobin is positively associated with MCHC in HVs, whereas there is no significant correlation between the two variables in patients with SCD, regardless of hydroxyurea status, based on the significant group interactions shown in Table 1 and the slope estimates shown in Table 2 (Figure 3(a): see statistically significant positive relationship between hemoglobin and MCHC in the healthy group and no relationship for the either patient group). Similarly, RBC counts are negatively associated with MCHC in untreated SCD patients, while no relationship is evident between the two in HVs (Table 1, Figure 3(b), and Table 2). The relationship between MCHC and RBC counts remains negative in patients with SCD treated with hydroxyurea but is not statistically significant (Figure 3(b) and Table 1). MCV is not significantly associated with MCHC in HVs or in SCD patients not taking hydroxyurea (data not shown). The comparison of estimated slopes indicates an alteration in the relationship between MCHC and hemoglobin concentration in patients with SCD, although the difference is slightly blunted in SCD patients taking hydroxyurea compared to those who are not (Table 2). A significant difference in the relationship between MCHC and RBC count is also confirmed in patients with SCD compared to HVs (Tables 1 and 2).

Since MCHC is influenced by the hydration status of the cell in addition to the amount of hemoglobin within it, we also analyzed each of the above parameters for correlations with mean corpuscular hemoglobin (MCH). As shown by the heat map (Supporting Figure 6), the strength and direction of correlations between all parameters and MCHC are largely maintained if MCH, which is not influenced by the hydration status of the cell, is used rather than MCHC.

### 3.4. Markers of Hemolysis and Reticulocyte Counts Are Associated With Iron Parameters in SCD Patients

**Figure 4:**
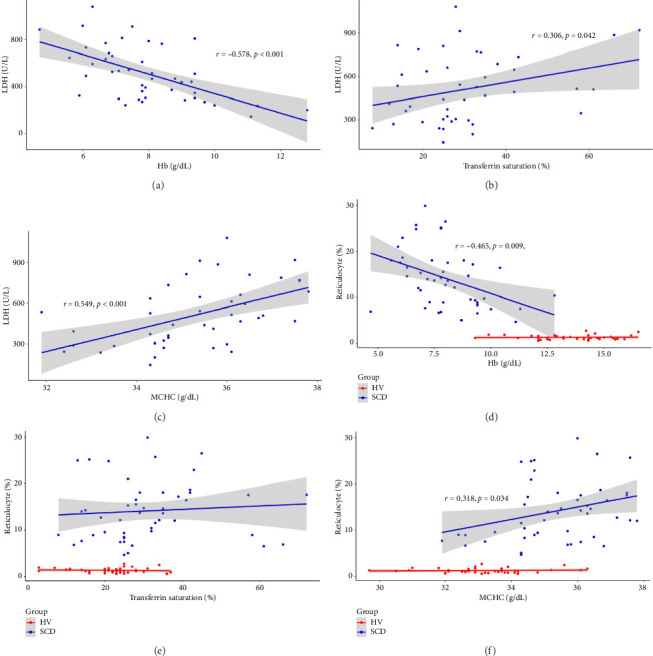
Relationship between indicators of hemolysis and hematological or iron parameters in patients with sickle cell anemia and healthy volunteers. Correlations demonstrate relationships between lactate dehydrogenase and (a) hemoglobin, (b) transferrin saturation, and (c) mean cellular hemoglobin concentration in patients with sickle cell disease (blue squares; *n* = 48). The relationship between reticulocyte percentage and (d) hemoglobin, (e) transferrin saturation, and (f) mean cellular hemoglobin concentration in patients with sickle cell disease (blue squares; *n* = 48) and healthy volunteers (red circles; *n* = 67) is also demonstrated by correlation.

Since the results from correlational analyses demonstrate altered relationships between iron status and hematological parameters in SCD, we investigated the relationships between markers of iron status and the hemolytic marker, lactate dehydrogenase. We also investigated the relationship between markers of iron status and reticulocyte percentage. Results indicate the expected inverse relationship between hemoglobin concentration and lactate dehydrogenase in patients with SCD (Figure 4(a)). Transferrin saturation (Figure 4(b)) and MCHC (Figure 4(c)) are both positively correlated with lactate dehydrogenase in patients with SCD. Lactate dehydrogenase measures are not available for HVs, as it is not routinely ordered. Reticulocyte percentage is also inversely associated with hemoglobin concentration in patients with SCD as expected (Figure 4(d)). While there is not a significant relationship between transferrin saturation and reticulocyte percentage in patients with SCD (Figure 4(e)), there is a positive association between MCHC and reticulocyte percentage (Figure 4(f)). Reticulocyte percentages are very low in HVs and are not significantly associated with these markers of iron status.

## 4. Discussion

Collectively, these data evidence specific relationships between iron status, MCHC, and severity of hemolysis in patients with SCD and provide support for a model where increased transferrin saturations contribute to increased intracellular concentrations of sickle HbS and worsening anemia in SCD. The correlation between MCHC and markers of hemolysis in SCD subjects provides *in vivo* support for the “delay time hypothesis” and is consistent with *ex vivo* studies showing a greater propensity of cells with higher hemoglobin concentrations to sickle [22, 23]. The positive correlation between MCHC and lactate dehydrogenase supports a mechanism whereby erythrocytes with lower concentrations of HbS are less susceptible to sickling and hemolysis [13, 24]. Importantly (with the caveat that inflammation may exert effects on transferrin saturation), the inverse correlation between transferrin saturation and RBC count and the positive correlation between transferrin saturation and lactate dehydrogenase each support the proposal that restricting serum iron may be effective in improving hematological parameters in patients with SCD. The influence of inflammation on transferrin saturation is complex. Although serum iron decreases in response to acute inflammation [25], transferrin itself is a negative acute phase reactant [26]. The effects may therefore cancel each other, resulting in no appreciable change in transferrin saturation.

Relationships between hematological parameters and iron status per se have not previously been explored in a defined cohort of patients with SCD. The data presented herein provide clinical support for preclinical studies suggesting mild iron restriction may be beneficial in the context of SCD [6–9]. Understanding these relationships has the potential to support and inform the development of iron-restricted therapeutic approaches in the treatment of SCD. Since limiting dietary iron intake is unlikely to be a practical means of antisickling therapy in patient management, the use of pharmacologic means to decrease circulating iron warrants continued investigation. To this end, a clinical trial investigating the utility of the ferroportin inhibitor vamifeport is currently recruiting subjects and pharmacologic agents targeting other iron regulatory molecules are under investigation.

## Figures and Tables

**Table 1:** Regression model summaries.

	Dependent variable			
	MCHC		Tf sat	
	∼Hb	∼RBC	∼Hb	∼RBC
	1	2	3	4
Intercept	27.018 (1.446)⁣^∗∗∗^	31.432 (1.538)⁣^∗∗∗^	0.543 (0.497)	1.733 (0.502)⁣^∗∗∗^
Hb	0.454 (0.107)⁣^∗∗∗^		0.181 (0.037)⁣^∗∗∗^	
RBC		0.363 (0.326)		0.272 (0.106)⁣^∗^
Group (SCD vs. HV)	9.415 (1.722)⁣^∗∗∗^	6.598 (1.762)⁣^∗∗∗^	3.185 (0.591)⁣^∗∗∗^	2.341 (0.575)⁣^∗∗∗^
Group (SCD_HU vs. HV)	6.924 (1.765)⁣^∗∗∗^	5.409 (1.762)⁣^∗∗^	3.200 (0.696)⁣^∗∗∗^	2.579 (0.575)⁣^∗∗∗^
Hb: Group (SCD vs. HV)	−0.591 (0.156)⁣^∗∗∗^		−0.218 (0.054)⁣^∗∗∗^	
Hb: Group (SCD_HU vs. HV)	−0.293 (0.153)		−0.183 (0.053)⁣^∗∗∗^	
RBC: Group (SCD vs. HV)		−1.385 (0.456)⁣^∗∗^		−0.517 (0.149)⁣^∗∗∗^
RBC: Group (SCD_HU v HV)		−0.936 (0.471)⁣^∗^		−0.510 (0.154)⁣^∗∗^
Observations	173	173	173	173
F statistic (df = 5; 167)	29.211⁣^∗∗∗^	26.771⁣^∗∗∗^		
Null deviance (df = 172)			50.425	50.425
Residual deviance (df = 167)			31.574	32.352

⁣^∗^*p* < 0.05.

⁣^∗∗^*p* < 0.01.

⁣^∗∗∗^*p* < 0.001.

**Table 2:** Estimated slopes of correlations.

Model	Variable	Group	Slope estimate	SE	*p* value	CI
MCHC (g/dL) and Tf sat (%) (Table 2)	Tf sat (%)	HV	0.082	0.021	< 0.001	0.04–0.12
	Tf sat (%)	SCD	0.048	0.013	< 0.001	0.02–0.07
	Tf sat (%)	SCD_HU	0.014	0.008	0.067	0.00–0.03

MCHC (g/dL) and Hb (g/dL) (Table 1, Model 1)	Hb (g/dL)	HV	0.454	0.107	< 0.001	0.25–0.66
	Hb (g/dL)	SCD	−0.136	0.114	0.233	−0.36–0.09
	Hb (g/dL)	SCD_HU	0.161	0.11	0.142	−0.05–0.38

MCHC (g/dL) and RBC (×10^6^/*μ*L) (Table 1, Model 2)	RBC (×10^6^/*μ*L)	HV	0.363	0.326	0.266	−0.28–1.00
	RBC (×10^6^/*μ*L)	SCD	−1.022	0.319	0.001	−1.65–0.40
	RBC (×10^6^/*μ*L)	SCD_HU	−0.574	0.34	0.091	−1.24–0.09

Tf sat (%) and Hb (g/dL) (Table 1, Model 3)	Hb (g/dL)	HV	3.734	0.837	< 0.001	2.09–5.30
	Hb (g/dL)	SCD	−1.129	1.229	0.358	−3.54–1.28
	Hb (g/dL)	SCD_HU	−0.073	1.565	0.963	−3.14–2.99

Tf sat (%) and RBC (×10^6^/*μ*L) (Table 1, Model 4)	RBC (×10^6^/*μ*L)	HV	5.567	2.240	0.013	1.18–9.96
	RBC (×10^6^/*μ*L)	SCD	−7.624	3.333	0.022	−14.16–1.09
	RBC (×10^6^/*μ*L)	SCD_HU	−9.887	4.674	0.034	−19.05–0.73

**Table 3:** MCHC and transferrin saturation linear regression model summary.

	Dependent variable
	MCHC
Intercept	31.454 (0.451)⁣^∗∗∗^
Tf sat	0.082 (0.021)⁣^∗∗∗^
Group (SCD vs. HV)	2.402 (0.623)⁣^∗∗∗^
Group (SCD_HU vs. HV)	3.358 (0.577)⁣^∗∗∗^
Tf sat: group (SCD vs. HV)	−0.035 (0.024)
Tf sat: group (SCD_HU vs. HV)	−0.068 (0.022)⁣^∗∗^
Observations	173
*F* statistic	33.104⁣^∗∗∗^ (df = 5.167)

⁣^∗^*p* < 0.05.

⁣^∗∗^*p* < 0.01.

⁣^∗∗∗^*p* < 0.001.

## Data Availability

Data described in this manuscript will be made available upon request pending application and approval.

## Supporting Information

Supporting Information 1 Supporting Table 1: Summary of subject characteristics stratified by cohort.

Supporting Information 2 Supporting Figure 1: Data filtering scheme.

Supporting Information 3 Supporting Figure 2: Hematologic parameters of healthy volunteers and patients with sickle cell disease with and without hydroxyurea treatment.

Supporting Information 4 Supporting Figure 3: Transferrin saturations of healthy volunteers and patients with sickle cell disease with and without hydroxyurea treatment.

Supporting Information 5 Supporting Figure 4: Comparison of fetal hemoglobin and markers of hemolysis in patients with sickle cell disease with and without hydroxyurea treatment.

Supporting Information 6 Supporting Figure 5: Relationships between fetal hemoglobin and transferrin saturation in patients with sickle cell anemia.

Supporting Information 7 Supporting Figure 6: Heatmap comparing relationships between MCH or MCHC with selected parameters in healthy volunteers or patients with sickle cell anemia.
