# Supplementary material for: Relationships Between Markers of Iron Status and Hematological Parameters in Patients With Sickle Cell Disease
Source: Adv Hematol. 2024 Dec 3;2024:9872440. doi: 10.1155/ah/9872440 (PMC11631288; doi:10.1155/ah/9872440)
Supplement: Supporting Information 1 — Supporting Table 1: Summary of subject characteristics stratified by cohort. [file 9872440.f1.docx]

**Supplementary Figure legends.**

**Supplementary Figure 1. Data filtering scheme.** Flow sheet describing the data filtering scheme utilized to identify patients meeting criteria for analysis.

**Supplementary Figure 2. Hematologic parameters of healthy volunteers and patients with sickle cell disease with and without hydroxyurea treatment.** Individual values of A) hemoglobin concentrations, B) red blood cell counts, C) hematocrit, D) mean cellular hemoglobin, E) mean cellular volume and F) mean cellular hemoglobin concentration from healthy volunteers (n=58), patients with sickle cell disease not taking hydroxyurea (n=48) and patients with sickle cell disease taking hydroxyurea (n=67) are presented. Average values are indicated with a horizontal bar

**Supplementary Figure 3. Transferrin saturations of healthy volunteers and patients with sickle cell disease with and without hydroxyurea treatment.** Individual values of transferrin saturation are presented for healthy volunteers (n=58), patients with sickle cell disease not taking hydroxyurea (n=48) and patients with sickle cell disease who are taking hydroxyurea (n=67). Average values are indicated with a horizontal bar.

**Supplementary Figure 4. Comparison of fetal hemoglobin and markers of hemolysis in patients with sickle cell disease with and without hydroxyurea treatment.** Individual values of A) fetal hemoglobin, B) percentage of reticulocytes and C) lactate dehydrogenase are presented for patients with sickle cell disease who are not taking hydroxyurea (n=48) and those who are (n=67). Average values are indicated with a horizontal bar.

**Supplementary Figure 5. Relationships between fetal hemoglobin and transferrin saturation in patients with sickle cell anemia.** Correlation between fetal hemoglobin and transferrin saturation in patients with sickle cell anemia who are not taking hydroxyurea (n=48).

**Supplementary Figure 6. Heatmap comparing relationships between MCH or MCHC with select parameters in healthy volunteers or patients with sickle cell anemia.** Heatmap comparing correlation coefficients of mean cellular hemoglobin and mean cellular hemoglobin concentration with hematologic or iron parameters in healthy volunteers and patients with sickle cell anemia.

Supplementary Table 1. Table 1. Summary of subject characteristics stratified by cohort. Data are presented as mean (SD) except for sex, which is presented as number(percentage) of males.

|  | **HV** | **SCD** | **SCD_HU** |
| --- | --- | --- | --- |
| **n** | 58 | 48 | 67 |
| **Age at collection (yrs)** | 34.29 (13.18) | 32.98 (9.59) | 35.01 (10.61) |
| **Sex = Male (%)** | 30 (51.7) | 25 (52.1) | 39 (58.2) |
| **MCHC (g/dL)** | 33.13 (1.30) | 35.34 (1.45) | 35.41 (1.23) |
| **MCV (fL)** | 86.73 (4.89) | 87.08 (7.40) | 104.72 (12.52) |
| **RBC (x10^6/uL)** | 4.69 (0.52) | 2.64 (0.58) | 2.49 (0.46) |
| **Hb (g/dL)** | 13.46 (1.55) | 8.03 (1.60) | 9.11 (1.40) |
| **Transferrin saturation (%)** | 20.45 (7.79) | 31.15 (14.07) | 41.46 (18.89) |

`
