# Supplementary figures and images for: Relationships Between Markers of Iron Status and Hematological Parameters in Patients With Sickle Cell Disease
Source: Adv Hematol. 2024 Dec 3;2024:9872440. doi: 10.1155/ah/9872440 (PMC11631288; doi:10.1155/ah/9872440)

## Parrow et al Supplementary Figure 1

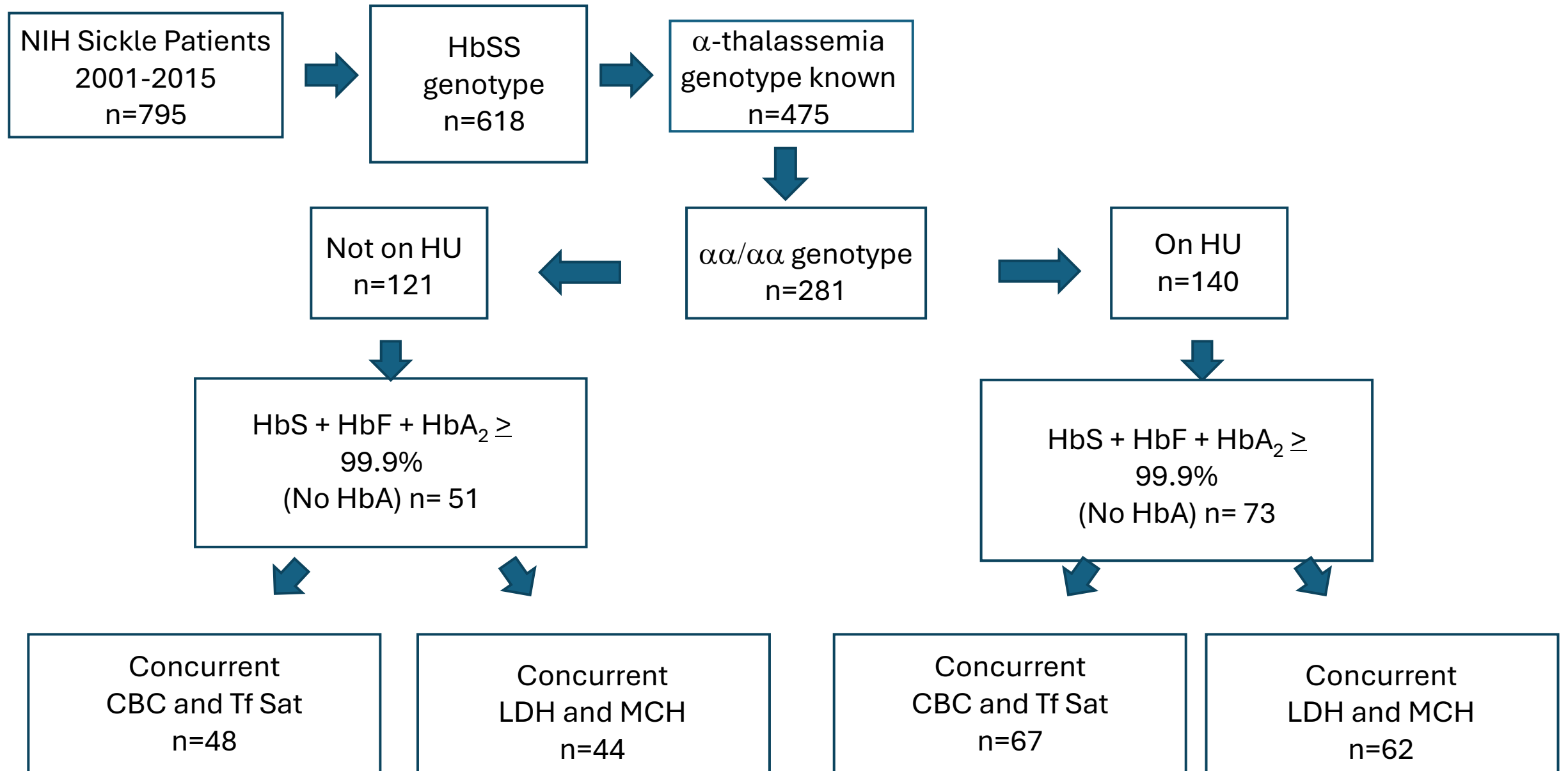

Supplement: Supporting Information 2 — Supporting Figure 1: Data filtering scheme. [file 9872440.f2.pdf]

Parrow et al Supplementary Figure 2

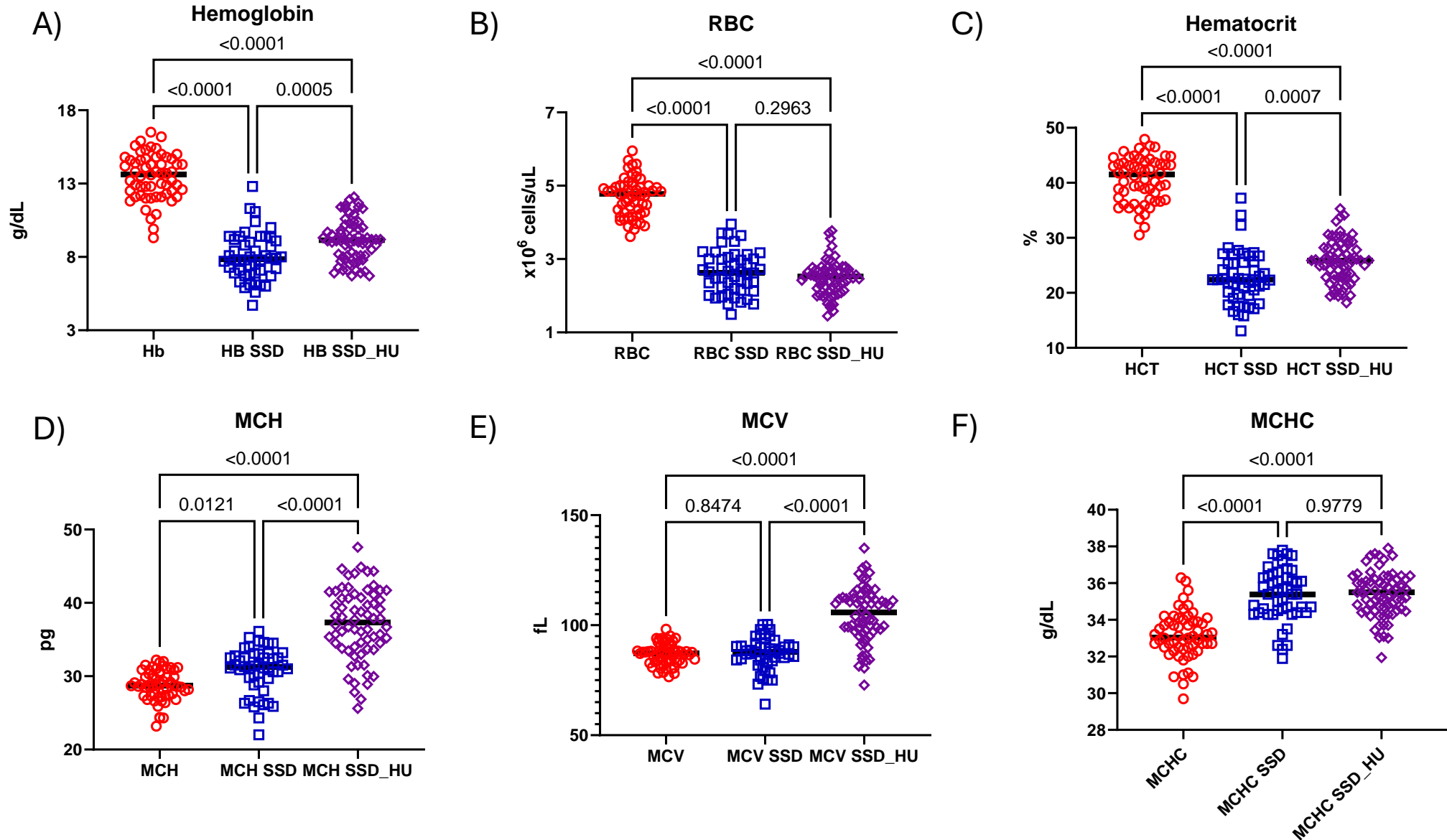

Supplement: Supporting Information 3 — Supporting Figure 2: Hematologic parameters of healthy volunteers and patients with sickle cell disease with and without hydroxyurea treatment. [file 9872440.f3.pdf]

Parrow et al Supplementary Figure 3

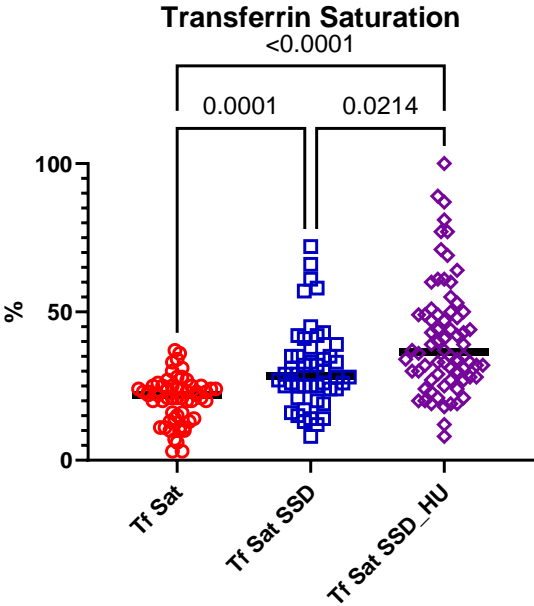

Supplement: Supporting Information 4 — Supporting Figure 3: Transferrin saturations of healthy volunteers and patients with sickle cell disease with and without hydroxyurea treatment. [file 9872440.f4.pdf]

Parrow et al Supplementary Figure 4

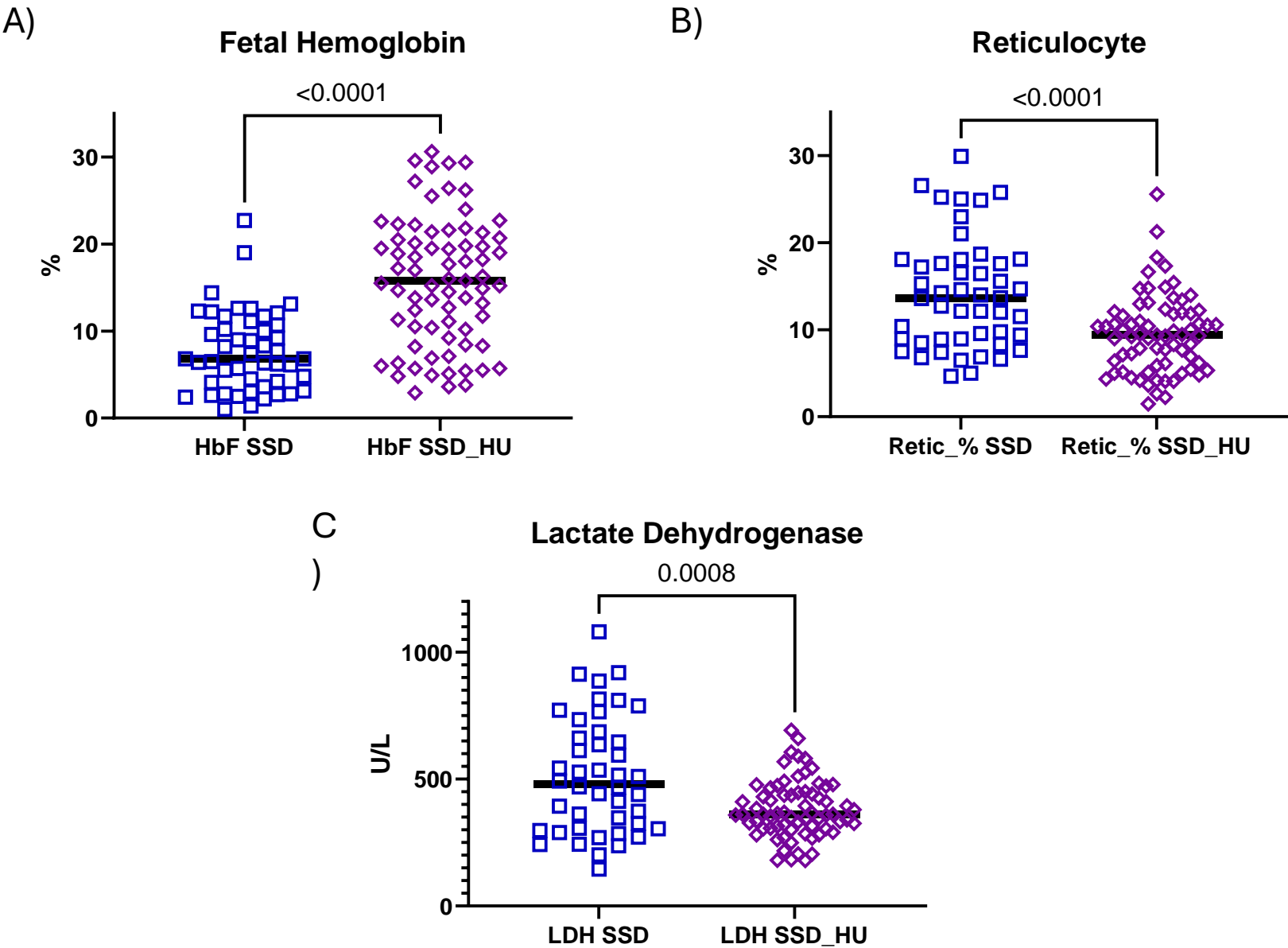

Supplement: Supporting Information 5 — Supporting Figure 4: Comparison of fetal hemoglobin and markers of hemolysis in patients with sickle cell disease with and without hydroxyurea treatment. [file 9872440.f5.pdf]

Parrow et al Supplementary Figure 5

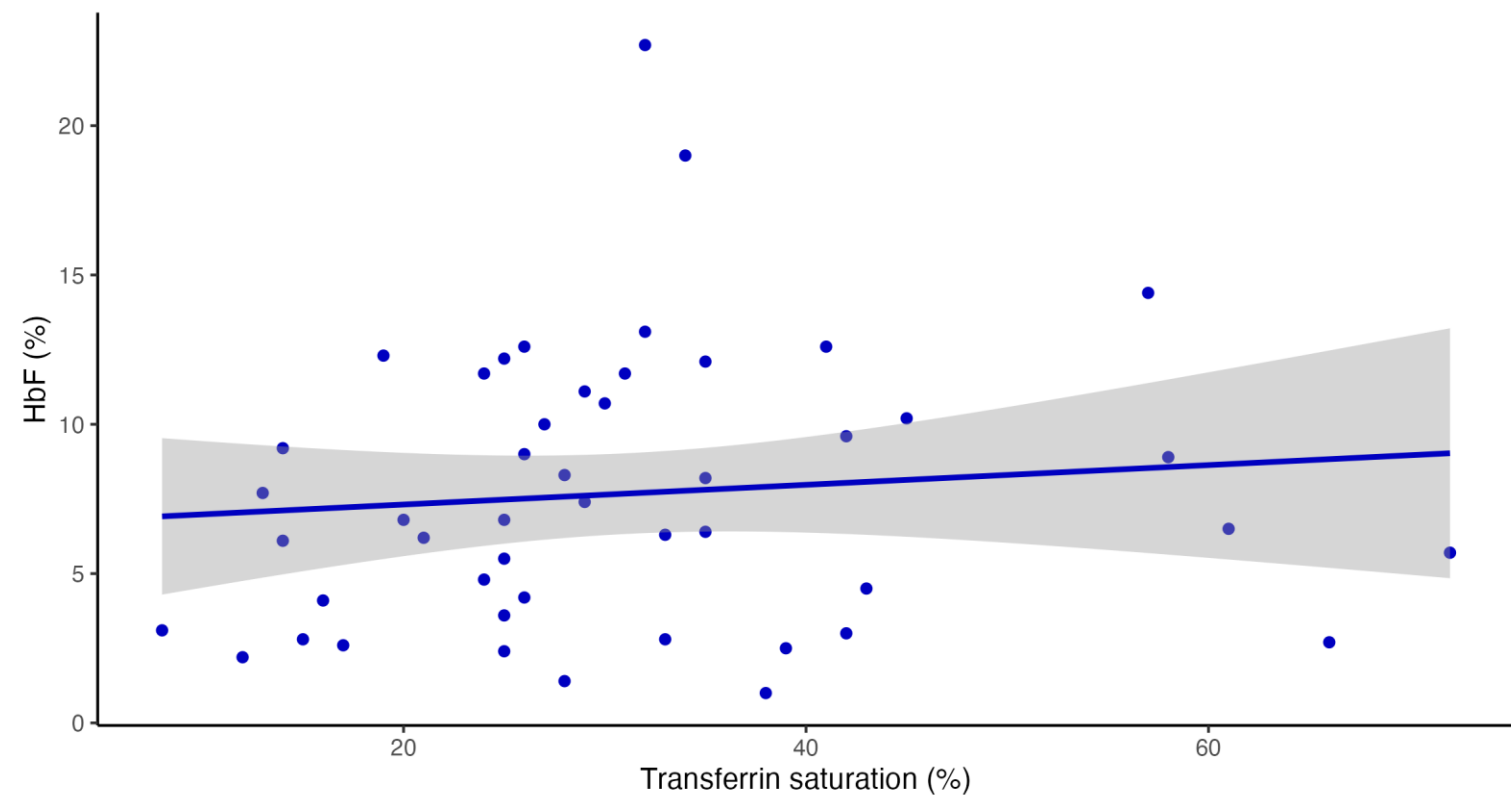

Supplement: Supporting Information 6 — Supporting Figure 5: Relationships between fetal hemoglobin and transferrin saturation in patients with sickle cell anemia. [file 9872440.f6.pdf]

Parrow et al Supplementary Figure 6

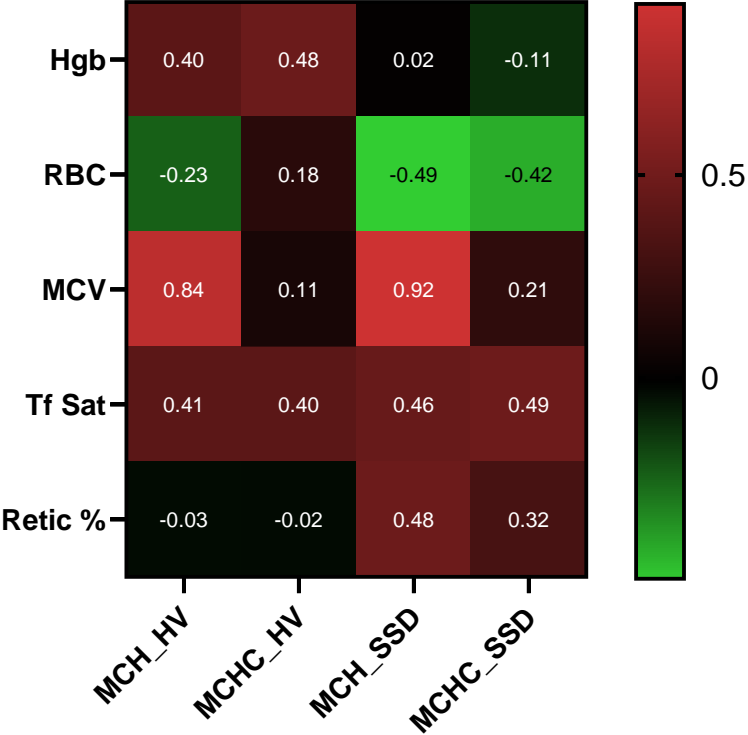

Supplement: Supporting Information 7 — Supporting Figure 6: Heatmap comparing relationships between MCH or MCHC with selected parameters in healthy volunteers or patients with sickle cell anemia. [file 9872440.f7.pdf]
